# Reality and challenges of peripheral vascular access: a mixed-methods study

**DOI:** 10.1016/j.ijnsa.2026.100554

**Published:** 2026-05-07

**Authors:** Laetitia Ricci, Florian Manneville, Côme Slosse

**Affiliations:** a CHRU-Nancy, Inserm, Université de Lorraine, CIC, Epidémiologie Clinique, Nancy, France; b Université de Lorraine, Inserm, INSPIIRE, F-54000, Nancy, France; c Anesthesiology and Critical Care Department, Nancy University Hospital Center, Nancy, France; d IADI, INSERM 1254, University of Lorraine, Nancy, Grand Est, France; e Université de Lorraine, Centre Universitaire d’Enseignement par Simulation (CUESIM), Nancy, France

**Keywords:** Vascular access devices, Difficult vascular access, Operating theater, Emergency department, Mixed methods, Algorithm, Implementation

## Abstract

**Background:**

While up to 60% of patients experience difficult intravenous access during peripheral venous catheter insertion, existing tools to prevent this phenomenon remain partial with limited integration of healthcare professionals' practical experience.

**Objective:**

To inform development of an algorithm (composed of a scale and a decision-making tree) tailored to puncture conditions for preventing failures and guiding responses when failures occur by identifying real needs and requirements of health professionals with respect to organization of peripheral venous catheter care.

**Design:**

Observational study with a parallel mixed methods approach.

**Setting(s):**

Two hospitals from northeastern France

**Participants:**

A total of 298 adult patients (quantitative data) and 17 healthcare professionals (qualitative data).

**Methods:**

Recruitment took place in an operating theatre and an emergency department in France. The study used a detailed description of existing peripheral venous catheter placement practices (auto-questionnaires) and individual semi-structured elicitations interviews with health professionals.

**Results:**

A total of 18% of patients had unsuccessful puncture on the first attempt. In cases of difficulties, health professionals mainly called other professionals for assistance. However, in a third of cases, they did not implement any strategy. During interviews, health professionals outlined variations in performance linked to patients’ characteristics and intraindividual factors (e.g., stress, fatigue, and emotional state). They emphasized the lack of specific placement protocols and called for them to be implemented. Factors related to health care setting emerged, such as i) working conditions (which do not always enable handover), ii) workspace layout, iii) difficulties in anticipating the workload, and iv) a lack of knowledge regarding nurses’ access to alternative techniques (e.g., transillumination and ultrasound).

**Conclusions:**

By identifying critical gaps in current catheter placement practices including the absence of standardized placement protocol and influence of contextual and emotional factors, this work will inform the development of a catheter placement algorithm.

**Registration:**

clinicaltrials.gov NCT05935228, registered 07/07/2023, first recruitment 05/12/2023.

What is already known

• Up to 60% of patients experience difficult intravenous access during peripheral venous catheters insertion.
• Existing tools to prevent difficult intravenous access, such as risk scores, remain partial with limited integration of health professionals’ practical experience.

What this paper adds

• Our findings reveal critical gaps in current practices particularly the absence of standardized placement protocols and the influence of contextual and emotional factors on procedural outcomes.
• This study identified practical and organizational levers that could enhance patient safety, reduce the number of punctures per patient, and support decision-making in high-pressure clinical environments.

Alt-text: Unlabelled box dummy alt text

## 1 Background

The parenteral route involves the administration of therapies via a device inserted into the patient's vascular system and is a recognized, safe, and effective therapeutic technique. With nearly two billion peripheral venous catheters inserted each year and 80% of patients admitted to the hospital, peripheral venous access is necessary in health care practice (Rickard et al., 2018; van Loon et al., 2018). Peripheral venous catheters affect many specialties and care techniques, which enable prevention, diagnosis, and treatment. Medical and allied health professionals primarily use the traditional or landmark visualization/palpation method to insert the catheter into the target vessel (Bodenham, 2017). However, there are situations such as difficult vascular access, in which this traditional technique fails to reach the target and puts the patient at risk of failed punctures and complications.

Difficult vascular access, which is not defined by consensus, affects between 12.6% and 59.3% of the population, depending on the study (Armenteros-Yeguas et al., 2017; Bahl et al., 2021; Civetta et al., 2019; Ng et al., 2022). The profiles of patients with difficult vascular access vary from congenital to acquired. Pathologies, such as obesity, drug addiction or a history of intravenous drug addiction, advanced age, cardiovascular pathologies or cancer, favor the development of difficult vascular access (Rodríguez-Calero et al., 2020). In addition to the patient profile, the environmental conditions and health professionals’ skills in the peripheral venous catheter are essential factors in the success of the procedure (van Loon et al., 2016, 2019). The consequences of difficult vascular access are varied, both individually and collectively. For example, difficult vascular access patients are at increased risk of local or systemic infection, venous thrombosis, nerve or skin injury, or psychological distress and trauma due to the multiple punctures caused by successive failures (Adhikari et al., 2010; Dinis and Sousa, 2023; Kinirons et al., 2000; Marsh et al., 2020; Rickard et al., 2018; Roditi et al., 2022; Stefanos et al., 2023; van Loon et al., 2018). Health professionals faced with failure may develop a sense of incompetence, with a risk of fatigue or even disgust with the profession and a changed self-image (Daloz, 2007). Finally, delays in treatment associated with difficult vascular access increase bottlenecks in care departments and costs to institutions (Haliday and Naudin, 2019).

The number of people potentially affected and the increased frequency of occurrence (due to the increase in the causes of difficult vascular access in the global population, led by obesity (NCD Risk Factor Collaboration, 2024) make difficult vascular access a significant public health issue.

Health professionals and researchers have proposed two main approaches to address the impact of this phenomenon: risk assessment and corrective measures. The first attempts to reduce the impact of difficult vascular access through upstream risk assessment with the creation of scores. The best known of these scoring systems is the modified A-DIVA score, developed by van Loon et al. (2019) (van Loon et al., 2019). The modified score is based on criteria considered important for the success of the procedure (visibility, palpability and diameter of the target vessel, history of difficult vascular access, and health professional experience). However, this tool is only a partial solution to the difficult vascular access problem as it does not address the issue. The second attempt to improve the management of patients with difficult vascular access involves developing decision trees, or algorithms, for grading the response to puncture failure, proposing alternative strategies to facilitate insertion (ultrasound, infrared) and thus structuring the organization of care (Ng et al., 2022; Stagg, 2023). However, the literature indicates that existing responses to puncture failure are incomplete (Ng et al., 2022; Stagg, 2023). In addition, the responses correspond to a priori constructions based on the literature, with little or no connection to the health professionals in the departments. Furthermore, no study has verified the effectiveness of the implementation of a decision-making tree tailored to puncture conditions (algorithm) for preventing and, if needed, grading the response to puncture failure. Finally, to the best of our knowledge, the two approaches (risk assessment vs. corrective action) have remained separate in their conceptualization and implementation. To address this gap, the present study – conducted within the framework of the “algorithm for peripheral venous catheter placement” (ALCOV) project (Slosse et al., 2024) – aimed to inform the development of an algorithm (composed of a scale and a decision-making tree) tailored to puncture conditions for preventing failures and guiding responses when failures occur by identifying real needs and requirements of health professionals with respect to organization of peripheral venous catheter care. The resulting algorithm is intended to be holistic and adaptable to both emergency and planned care, covering all relevant tools and devices.

## 2 Methods

### 2.1 Design

This study used data from Phase I of the ALCOV project and consists of the collection of (i) quantitative data on real practices on the ground and (ii) qualitative data on health professionals' views on peripheral venous catheters and difficult vascular access. This information is essential for identifying the real needs and requirements of health professionals with respect to the organization of peripheral venous catheter care. The aim of ALCOV was to evaluate the effect of implementing a peripheral venous catheter placement algorithm versus standard care on the mean number of punctures per patient. ALCOV is a mixed (quantitative and qualitative), multicenter, non-randomized, quasi-experimental, before-and-after, open-label, cross-sectional study with control and experimental groups consisting of three phases. Briefly, Phase I was observational and used a parallel mixed-method approach with an in-depth and detailed description of existing practices for peripheral venous catheter placement using auto-questionnaires (quantitative part) and individual semi-structured interviews with health professionals (qualitative part) using an elicitation interview design. The aim of the elicitation interview method is to encourage verbal description of the way a task is actually performed (Balas-Chanel, 2002; Vermersch, 2006, 2004), thus 'reliving' the subjective lived experience of a past, specific and unique situation (Mouchet et al., 2019). Phase I started in December 2023 and ended in July 2024. The ALCOV project protocol is fully detailed elsewhere (Slosse et al., 2024). The quantitative and qualitative results were presented separately but discussed in an integrated manner by three researchers from different disciplines (nurse anesthetist, psychologist, and epidemiologist).

### 2.2 Study setting and sampling

Recruitment took place at the University Hospital of Nancy (CHRU de Nancy) and the Pont-à-Mousson Hospital (Eastern France) and concerned health professionals and adult patients. The operating theatre at CHRU de Nancy performs care activities requiring the placement of 100–150 peripheral venous catheters per week (5000–7800 placements per year). In the Pont-à-Mousson emergency department, 881 peripheral venous catheters were placed in 3 months in 2018 (>3500 placements per year).

For the quantitative part, auto-questionnaires were completed by health professionals (nurses, anesthetic nurses, and physicians attached to the department) who punctured patients. It was estimated that a total of 298 punctures will occur during phase I of the ALCOV project (Slosse et al., 2024).

For the qualitative part, we applied a maximum variation sampling strategy using the participants’ profession as the main criterion of variation (nurse, specialized nurse (anesthetic nurse) and physician) attached to each of the two departments. The principal investigator of each of the two departments (CS, JCO) selected health professionals who met our sampling criteria and invited them to participate. None refused. The project manager (AO) then contacted the identified health professionals to arrange an interview. We estimated the sample size on the basis of the literature without assessing and reporting thematic saturation using a calculation approach such as that proposed by Guest et al. (Guest et al., 2020).

Data saturation is generally defined as the point at which the added value of conducting further interviews becomes minimal, as no new or substantially different findings emerge. A sample size between 11 and 20 interviews is generally considered appropriate in qualitative studies to achieve thematic saturation (Blaikie, 2018). Given the challenges in providing clear evidence to support claims of data saturation, the concept of data sufficiency based on information power has emerged (Malterud et al., 2016). This approach, while grounded in the rigor inherent to qualitative research, depends on the researchers’ aims; for example, studies aiming to describe a phenomenon may require a smaller number of interviews than those seeking to develop theory (LaDonna et al., 2021).

### 2.3 Elicitation interview guides

A health psychologist (LR) specializing in qualitative methods proposed the first draft of the interview guide. The scientific team project, consisting of a PhD anesthetic nurse (CS), a sociologist (CF) and an epidemiologist (FM), then revised and stabilized the guides during a two-hour meeting (Supplementary material).

### 2.4 Data collection

For the quantitative part, the auto-questionnaire assessed patient characteristics (age [year], sex [male/female], whether the patient was perceived as difficult to puncture before the puncture [yes/no] from both the patient's and the health professional's perception, and whether the health professional perceived the patient as difficult to puncture after the puncture [yes/no]), health professionals characteristics (workstation of the main and secondary health professionals [daytime/night/week-end], function of the main and secondary health professionals [nurse/ anesthetic nurse/physician], years of experience of the main and secondary health professionals [<1 year/1–3 years/3–5 years/5–10 years/>10 years], puncture data [number of punctures needed [1/2/3/4/5], number of health professionals required [1/2/3/>3], final success of punction (yes/no), difficulties encountered by health professionals (yes/no), first and second (if applicable) strategies in case of difficulties (calling another professional/use of an alternative puncture technique/choice of another type of vascular type of vascular/withdrawal/other/no strategy implemented), and pain data measured immediately after puncture (pain scoring [yes/ no], and pain score using numerical scale ranging from 0 [no pain] to 10 [maximum pain]).

For the qualitative part, the principles of the elicitation interview method are as follows: (i) focus the participant's attention on a single real situation (situated in time and space), (ii) be directive to encourage verbal elaboration of the procedural dimension, (iii) encourage specification of the context of the situation, and (iv) use open-ended questions to encourage description of the action (what, when, where and how). A two-year-experienced sociologist with an MSc degree (CF) conducted the interviews, either by videoconference (Microsoft Teams®) or by phone or face-to-face, at the discretion of the participants from December 2023 to February 2024. Each interview lasted between 45 min and 1 h. No one else was present during the time of the interview.

### 2.5 Data processing and analysis

Patient and health professionals’ characteristics, puncture data, and pain scores are presented as medians (interquartile ranges) for quantitative variables and as numbers (%) for categorical variables. These data are described overall and by center. The perception of the patient as difficult to puncture before the puncture was cross-tabulated with the health professional's perception, assessed both before and after the puncture.

A service provider subject to a strict confidentiality agreement transcribed anonymously the audio records from the interviews into text. The provider was selected specifically for her extensive experience in qualitative research transcription (e.g., any unclear or inaudible segments were explicitly indicated in the transcripts, ensuring transparency regarding potential uncertainties in the data)

Following an inductive approach (Chapman et al., 2015), from the first three interviews, the health sociologist and a M.D. student (MEA) developed the first coding grid, which underpinned the thematic analysis of the data. The health sociologist who conducted the interviews reviewed the transcripts during the coding process. This step allowed for an additional level of verification, as familiarity with the original interviews facilitated the identification and clarification of any ambiguities that may have arisen during transcription.

The other members of the scientific project team (a PhD anesthetic nurse, a psychologist and an epidemiologist) then modified the proposal, adding details, clarifications and suggestions to arrive at exhaustive and mutually exclusive categories of themes. The coding process thus involved researcher triangulation involving the perspectives of a health psychologist, a sociologist and a clinician/epidemiologist, providing a rigorous methodological framework to provide a deeper and broader perspective on the data analyses (Halcomb and Andrew, 2005).

### 2.6 Rigor

CF and MEA were double coded for each interview. The two coders resolved all coding disagreements by discussion to reach a Cohen’s kappa coefficient value of at least 0.80. Data analysis was conducted with NVivo v11.

### 2.7 Ethical statement

In accordance with Article L.1123–6 of the Public Health Code, the research Public Health Code, the research protocol was submitted by the promoter to the Institutional Review Committee (Comité de Protection des Personnes (CPP)). The study and associated informed consent were approved by CPP Sud-Méditerranée I on 25 April 2023 with reference number 2023-A00223–42. The study was registered at clinicaltrials.gov under the trial number NCT05935228. This study was supported by a grant from the French Eastern Interregional Group of Clinical Research and Innovation (GIRCI Est) (Appel à Projet PARAmédical, APPARA 2022) and by CHRU de Nancy. All the participants provided verbal consent to participate, including the publication of anonymous quotes.

## 3 Results

### 3.1 Patient and health professionals’ characteristics, puncture data, and pain scores

A total of 298 (149 per center) patients were included and a total of 375 punctures were realized. The patients were 60 (41–73) years old, 157 (53%) were female, and they reported, before the puncture, that they were just as difficult to puncture (n = 66; 22%) as the health professionals estimated (n = 63; 21%) (Table 1). After puncture, health professionals found that 39 patients (14.0%) had difficulty with puncturing. Health professionals in Centre 1 tended to report more patients as difficult to puncture than those in Centre 2 did, both before and after puncture. Overall, 58.5% of patients who perceived themselves as difficult to puncture before the puncture were also perceived as difficult to puncture by health professionals. The prevalence was 25.4% according to health professionals’ perceptions after puncture. Almost all the patients had puncture performed during the daytime (n = 269, 91%) by nurses (n = 289, 100%), and the majority of health professionals had >10 years of experience (n = 177, 60%). Health professionals’ characteristics were similar across centers. A total of 245 (82%) patients had a successful puncture at the first attempt, and the vast majority of patients required only one health professional (n = 283, 95%). Health professionals reported difficulties in 39 (14%) patients, which were more common in Centre 1 (n = 28, 20%) than in Centre 2 (n = 11, 8.5%). In these cases, health professionals called mainly other health professionals (n = 13, 33%) or did not implement any strategy (n = 13, 33%). Pain was scored in 169 (n = 56.7%) patients, with a median of 0.0 (0.0–3.0) points.

**Table 1:** Description of patients’ and health professionals’ characteristics, puncture data, and pain scores (n = 298 punctures).

	Total (n = 298)		Centre 1 (n = 149)		Centre 2 (n = 149)	
	n	%	n	%	n	%
**Patients’ characteristics**						
Age (year), median IQR	60	41–73	60	44–70	62	40–76
Missing	1		0		1	
Sex (male)	140	47.0	64	43.0	76	51.0
Missing	1		0		1	
Patient difficult to prick before puncture (yes)						
Patient perception	66	22.0	33	22.0	33	22.0
Missing	2		0		2	
Health professional perception	63	21.0	38	26.0	25	17.0
Missing	4		0		4	
Patient difficult to prick after puncture (health professional perception) (yes)	39	14.0	28	20.0	11	8.5
Missing	26		6		20	
**Health professionals’ characteristics**						
Workstation of the main health professional						
Daytime	269	91.0	130	88.0	139	93.0
Night	12	4.1	3	2.0	9	6.0
Week-end	15	5.1	14	9.5	1	0.7
Missing	2		2		0	
Function of the main health professional						
Nurse	289	100.0	147	99.0	142	100.0
Anesthetic nurse	1	0.3	1	0.7	0	0.0
Physician	0	0.0	0	0.0	0	0.0
Missing	8		1		7	
Years of experience of the main health professional						
< 1 year	16	5.4	0	0.0	16	11.0
1–3 years	10	3.4	0	0.0	10	6.8
3–5 years	5	1.7	3	2.0	2	1.4
5–10 years	89	30.0	53	36.0	36	24.0
> 10 years	177	60.0	93	62.0	84	57.0
Missing	1		0		1	
Workstation of the secondary health professional						
Daytime	6	86.0	5	83.0	1	100.0
Night	0	0.0	0	0.0	0	0.0
Week-end	1	14.0	1	17.0	0	0.0
Missing	291		143		148	
Function of the secondary health professional						
Nurse	6	86.0	5	83.0	1	100.0
Anesthetic nurse	0	0.0	0	0.0	0	0.0
Physician	1	14.0	1	17.0	0	0.0
Missing	291		143		148	
Years of experience of the secondary health professional						
< 1 year	0	0.0	0	0.0	0	0.0
1–3 years	1	14.0	1	17.0	0	0.0
3–5 years	1	14.0	1	17.0	0	0.0
5–10 years	4	57.0	4	67.0	0	0.0
> 10 years	1	14.0	0	0.0	1	100.0
Missing	291		143		148	
**Puncture data**						
Number of punctures needed						
1	245	82.0	118	79.0	127	85.0
2	37	12.0	22	15.0	15	10.0
3	10	3.4	6	4.0	4	2.7
4	4	1.3	1	0.7	3	2.0
5	2	0.7	2	1.3	0	0.0
Missing	0		0		0	
Number of punctures needed, median IQR	1.0	1.0–1.0	1.0	1.0–1.0	1.0	1.0–1.0
Missing	0		0		0	
Number of health professionals required						
1	283	95.0	141	95.0	142	95.0
2	11	3.7	6	4.0	5	3.4
3	3	1.0	1	0.7	2	1.3
>3	1	0.3	1	0.7	0	0.0
Missing	0		0		0	
Final success of punction (yes)	295	99.0	149	100.0	146	99.0
Missing	1		0		1	
Difficulties encountered by health professionals (yes)	39	14.0	28	20.0	11	8.5
Missing	26		6		20	
First strategy in case of difficulties						
Calling another professional	13	33.0	8	29.0	5	45.0
Use of an alternative puncture technique	1	2.6	0	0.0	1	9.1
Choice of another type of vascular access	5	13.0	4	14.0	1	9.1
Withdrawal	1	2.6	0	0.0	1	9.1
Other	6	15.0	5	18.0	1	9.1
No strategy implemented	13	33.0	11	39.0	2	18.0
Missing	0		0		0	
Second strategy in case of difficulties						
Calling another professional	3	33.0	0	0.0	3	33.0
Use of an alternative puncture technique	2	22.0	0	0.0	2	22.0
Choice of another type of vascular access	1	11.0	0	0.0	1	11.0
Withdrawal	1	11.0	0	0.0	1	11.0
Other	0	0.0	0	0.0	0	0.0
No strategy implemented	2	22.0	0	0.0	2	22.0
Missing	0		0		0	
**Pain**						
Pain scoring (yes)	173	58.0	67	45.0	106	72.0
Missing	2		0		2	
Pain score, median IQR	0.0	0.0–3.0	0.0	0.0–3.0	1.0	0.0–3.0
Missing	6					

The data are presented as numbers and percentages unless otherwise indicated. The percentages may not total 100 because of rounding.

### 3.2 Description of the characteristics of the interviewed health professionals

Table 2 shows that the sample was sufficiently diverse to gather a wide range of perspectives, as we recruited health professionals in two different departments with different profiles in terms of job, age and years of experience in their departments.

**Table 2:** Sociodemographic characteristics of interviewed participants (n = 17).

Code	Age	Length of years of service	Sex	Profession	Work place	Interview modality
Phys.Opera thea.W.1	32	1	Female	Physician	Operating theatre	Teams®
Anaes.RN.Opera thea.M.2	58	29	Male	Anesthetic nurse	Operating theatre	Face to face
Nurse.Opera thea.W.3	28	6	Female	Nurse	Operating theatre	Teams®
Phys.Opera thea.W.4	48	17	Female	Physician	Operating theatre	Phone
Nurse.Opera thea.W.5	28	6	Female	Nurse	Operating theatre	Teams®
Nurse.Opera thea.W.6	45	7	Female	Nurse	Operating theatre	Teams®
Nurse.Opera thea.W.7	36	2	Female	Nurse	Operating theatre	Teams®
Anaes.RN.Opera thea.M.8	56	17	Male	Anesthetic nurse	Operating theatre	Teams®
Anaes.RN.Opera thea.M.9	47	15	Male	Anesthetic nurse	Operating theatre	Phone
Nurse.Emergency.W.10	31	1	Female	Nurse	Emergency department	Phone
Nurse.Emergency.W.11	23	1	Female	Nurse	Emergency department	Face to face
Nurse.Emergency.M.12	45	2	Male	Nurse	Emergency department	Face to face
Nurse.Emergency.W.13	27	3	Female	Nurse	Emergency department	Face to face
Nurse.Emergency.M.14	25	13	Male	Nurse	Emergency department	Face to face
Nurse.Emergency.W.15	46	9	Female	Nurse	Emergency department	Face to face
Nurse.Emergency.W.16	54	10	Female	Nurse	Emergency department	Face to face
Nurse.Emergency.W.17	32	10	Female	Nurse	Emergency department	Face to face

We extracted five main themes from the inductive data analysis of the elicitations interview: (i) difficult intravenous access, (ii) training courses, (iii) organization for peripheral venous catheter placement, (iv) tips and areas where improvements can be made, and (v) impact of vascular access failure on health professionals (See Table 3 for a detailed description of the thematic analysis).

**Table 3:** Description of the thematic analysis of the elicitation interviews with health professionals.

Theme	Sub-theme	Main findings
**Health professionals’ perceptions of difficult intravenous access**	Incidence and risk factors	No consensus on the incidence of difficult vascular access. Numerous patient profiles identified as potential risk factors. Importance of human factors in peripheral venous catheter placement. Intra-professional variability in success depending on non-identified factors.
	Anticipation and management	Need for planning ahead peripheral venous catheter placement and for more anticipation to better adapt to the situation. Difficult vascular access is perceived as a professional risk that always requires solutions, with systematic attempts to find answers.
**Training courses**	Initial training	Fundamental basis for learning peripheral venous catheter placement, but limited by lack of practice and reliance on accompaniment, generating inequalities between students. Considered insufficient and dependent on informal clinical learning. Practices become progressively harmonized over time, relatively independent of years of experience.
	Specific training	Strong demand for training in alternative techniques (transillumination, ultrasound), perceived as beneficial for patients (less pain, fewer missed attempts) and professionals (less failure, skill development). Concerns remain regarding rights and opportunities for daily practice.
**Organization for managing peripheral venous catheter placement**	General hospital context	Negative impact of hospital productivity constraints on practice. Lack of visibility of departmental workload, creating operational difficulties.
	Department context	Organizational and spatial issues, especially for PICCs and midlines requiring coordinated placement. Lack of structured organization for these devices. Teamwork considered essential, but undermined by night and weekend understaffing, limiting solidarity.
	Guidelines	Need for best practice guidelines to optimize patient care in peripheral venous catheter placement.
**Tips and areas for improvement**	Individual professional level	Patient feedback during insertion provides key guidance. Numerous informal tips used in practice.
	Interprofessional informal level	Common practice of allowing two attempts before handover, with attention to patients’ physical and psychological well-being. Reliance on more experienced colleagues in case of failure.
	Department organization level	Lack of structured and formalized protocol for vascular access failure management.
**Impact of vascular access failure on health professionals**	Psychological and professional impact	Failure affects confidence in performing peripheral venous catheters and may impact overall professional self-confidence.

### 3.3 Health professionals’ perceptions of difficult intravenous access

- There is no consensus on the incidence of difficult vascular access.

Health professionals described numerous patient profiles as potential risk factors for difficult vascular access

Drug-addicted patients, obesity, amputated limbs, so the site is limited, mastectomy, bleeding, coming from mobile emergency and intensive care departments, a trapped arm, hypothermia. Nurse.Emergency.W.15

- Importance of human factors in the placement of peripheral venous catheters

Health professionals noted that there was intra-health professional variability in the success of peripheral venous catheter placement depending of non-identified factors.

It may be that you're not always consistent when it comes to performing and successfully inserting venous lines. Anaes.RN. Opera thea.M.8

Another very important thing I find when I'm inserting venous lines is that whenever I'm stressed, if I tell myself I'm going to miss something, I'm going to miss something. Nurse. Opera thea.W.5

- Need for planning ahead peripheral venous catheter placement

Health professionals call for more anticipation to allow better adaptation to the situation.

We have to warn them beforehand because we know that a drug-addicted patient whose veins have been injected intravenously will find it very complicated. That's why we don't insist too much on the peripheral venous, we quickly switch to central venous access under ultrasound. Anaes.RN. Opera thea.M.8

- difficult vascular access as a professional risk that always requires solutions

Health professionals find answers to any difficult vascular access problem.

It's a fairly common professional hazard, and one that we encounter time and time again. Anaes.RN. Opera thea.M.8

I've never felt helpless in a situation. You tell yourself there's always a solution (…), I find that in the end, you don't have too many options. It's rare that you really don't succeed. Nurse.Emergency.W.11

### 3.4 Training courses

- Initial training

For health professionals, initial training is a fundamental basis for learning about peripheral venous catheter placement.

With a right training, after two hours, you know how to play a peripheral venous catheter. But our job is to know why we do it, how we do it and to anticipate needs. But the act itself is a necessary element of everything else, (…) why we do it and what the consequences might be. An incorrectly placed line, or on a vein we don't really know, well, he goes for a scan, we inject a product, it bursts, so the product, which is obviously hyper-toxic, which is under the skin, burns. In this case, the catheter is inserted incorrectly or we've forced it in and well, people come out and then two days later they have a superficial thrombosis. Nurse.Emergency.W.16

However, health professionals emphasized the lack of practice in initial training and learning to place themselves essentially on the basis of accompaniment, which is a source of inequality between students.

When you get there and you've never practiced at the nurse training institutes, anywhere, for me every morning was a nightmare, I thought I'm going to fail, I'm going to fail, it was a disaster, so I think it's also the pressure that does it. Nurse.Opera thea.W.5

You have to be lucky to get internships where you can, where they let you take the risk when you're young, when you're at school. Anaes.RN.Opera thea.M.8

We have the trainees watch us several times so they can see how we do it and then we help them find the vein and then once they start to get stuck often, they try and then we take over again and then we help them little by little until they are completely autonomous on easier patients where we can see the vein spontaneously and until they are completely autonomous, for me it's a training program that doesn't take a day. It takes several days, several weeks to train a caregiver to place peripheral venous catheter. Nurse.Opera thea.W.6

Initial training is generally considered insufficient and highly dependent on opportunities for informal accompaniment throughout clinical practice. Finally, as training is largely shaped by departmental-level practices, clinical approaches tend to become relatively harmonized and homogenized among health professionals over the time, relatively independent of years of experience.

- Specific training

There is a real demand from nurses to access training in alternative techniques: transillumination and ultrasound, with the perception of benefits for patients (less pain, fewer missed opportunities), and themselves (less confrontation with failure, development of skills).

However, nurses expressed concerns about the rights and practice opportunities for them on a day-to-day basis

It's true that if there were more people trained in placement under ultrasound, it would be easier and less painful for patients. Nurse. Opera thea.W.7

Maybe if I'd been trained in ultrasound, I'd get the ultrasound machine out straight away and end up puncturing the patient just once instead of 3 or 4 times (…) I would like to be trained in ultrasound, but I don't know if the law gives us the right to be trained. Nurse. Opera thea.W.3

### 3.5 Organization for managing the placement of peripheral venous catheters

- General hospital context

Health professionals highlighted the negative impact of the hospital productivity context on their practices.

It takes us two seconds [to place a poor peripheral venous catheter], but if we look hard enough, it'll take us 15 min. If you spend 15 min with each patient, you see 4 of them every hour. In this age of hospital productivity, the math is quickly done. Nurse.Emergency.W.15

Health professionals underlined the lack of visibility of the level of frequentation in the departments, which can put them in difficulties.

We depend a great deal on the timing of surgery and the time it takes to discharge patients. There may well be one or 12, but it's difficult to know in advance how many patients will be in the operating theatre. The attendance rate is impossible to anticipate (…). We can be in a hurry for several reasons: because the patient doesn't have perfusion, because there have been several failures. He still needs to be operated on, so you have to work fast. The question of time can be stressful. Anaes.RN.Opera thea.M.8

- Department context

Health professionals describe organizational and space problems in departments, especially for peripherally inserted central catheters (PICCs) and ultrasound-guided long peripheral intravenous catheters (midlines), which must be placed in pairs.

We don't really have a dedicated area, we don't have a room that's a little bit separate from the operating theatre where we can set up in peace and quiet, under strict conditions of asepsis (…). If we had a dedicated area with the equipment in the same room, it's really more a question of ergonomics, it would save the nurse who's doing the set-up from having to run back and forth to get the equipment. Phys.Opera thea.W.1

What has not yet been organized is the placement of the midlines and PICCs. Anaes.RN. Opera thea.M.8

For health professionals, working as a team appeared essential to the success of peripheral venous catheter placement.

If a colleague is in difficulty, I go and help him and vice versa. There aren't too many problems between us either; there's always someone to come and help us out when we're in difficulty. Anaes.RN. Opera thea.M.2

[In case of failure] We're looking with both hands, we're both thinking about where. It's two brains, more than just technique… the technique is the same, we're equal, 100% equal in terms of skill and dexterity. We'll get there and maybe I'll even do it in the end, but in the end, we'll say 'oh, I've got a better feeling about this, what do you think? We're a team. Nurse.Emergency.W.15

However, they also emphasized that understaffing at night and weekends hinders solidarity between health professionals.

At night, for example, there's only one nurse, and sometimes you have to go and help her. Anaes.RN. Opera thea.M.8

During the week, it's good to have the other colleagues, the anesthetic nurse and the physicians. At weekends it's a small team, it's emergencies, you're not on your own at weekends. Nurse. Opera thea.W.3

- Guidelines

Health professionals stressed the need for best practice guidelines for peripheral venous catheter placement to optimize overall patient care.

Beyond the training, we need good practice guidelines. Elbow insertion - well, if a young arrives and can't find it, OK, but some people don't think too much about it. Green catheters instead of pink […]. Settling in is the same thing, people know they have to settle in, but if they don't want to, they don't want to. Nurse.Emergency. W10

Things change, products change, equipment changes. It's not always easy to know what to do, so the fact that you have a protocol clearly states which products and techniques are effective. Protocols can only be beneficial, they can also be a guide. Nurse. Opera thea.W.6

### 3.6 Tips and areas where improvements can be made

- At the health professional level

For health professionals, hearing the patient when placing a peripheral venous catheter provides valuable information to guide them.

Patients know when it's difficult to inject, so they say it's better to inject this arm or that arm. We listen to them because they know themselves. Nurse.Emergency. W10

In addition, health professionals often have many tips for placement.

There are several little tips. Apply a little alcohol [on the puncture site to facilitate vasodilation], warm the limb, compress the hand as if you were holding an anti-stress ball. Anaes.RN.Opera thea.M.9

- At the informal inter health professional level

In general, health professionals agree to allow themselves two attempts before handing over and keeping in mind patients’ physical and psychological well-being.

I set myself this barrier of two times. I feel that I don't want to hurt the patient, and then the relationship of trust, after two times, he will say to himself: a bit of stubbornness on me. Nurse. Opera thea.W.6

I tell myself two, three times maximum, and then I'll pass the baton. What's going to be left for the other person if I've taken from the fingers to the shoulder? You have to know how to hand over early on, otherwise you don't leave much chance for the next person and the patient. Nurse.Emergency.W.15

In cases of failure in vascular access placement, health professionals tend to rely on more experienced colleagues.

I’d look from someone I know experienced. Nurse.Emergency.W.15

Someone who perhaps doesn't have the same technique as me, who will know better how to perform and who also knows what is a complicated patient. Nurse. Opera thea.W.5

- At the department organization level

Health professionals underlined a lack of a structured and formalized response to vascular access failure protocolized in departments.

It's a bit like the way health professionals are organized. We say to each other: if you don't make it, call me. It's a bit like that. There's no dictated protocol; it's really between us, between colleagues (…). I missed once. My colleague missed a second time and a third time, she missed. We called a third person who came: it was a dud. The doctor finally took out his echo Doppler to get a good look at the veins, he missed. It ends up in foot. Nurse.Emergency.W.11

Personally, I stay with my colleague for the duration of the placement. If need be, I tell him that I've already tried this or that vein, that I've encountered difficulties. Phys.Opera thea.W.1

### 3.7 Impact of vascular access failure on health professionals

Vascular access failure can affect health professionals' confidence in their ability to perform peripheral venous catheters and even their confidence in their general professional skills.

That's what my colleagues and I always say: if you miss your first placement of the day, you won't make it the rest of the day. Nurse. Opera thea.W.5

When I first arrived and I wasn't used to placing, it was complicated. If you miss it, you say to yourself: it's a failure, you're no good, you have to move on. Nurse. Opera thea.W.3

## 4 Discussion

The aim of Phase I of the ALCOV project was to provide data on difficult vascular access and associated corrective actions already implemented on the basis of auto-questionnaires (quantitative part) and individual semi-structured interviews with health professionals (qualitative part).

Among the included patients, 22% thought they were difficult to prick, whereas 21% of the patients receiving peripheral venous catheters were identified as at risk from the point of view of health professionals. In both pre-evaluations, the predictions were concordant in just over half of the cases. These data highlight the unreliability of subjective interpretation concerning the anticipation of risks and raise the question of the relevance of the following two items included in the A-DIVA score: history of vascular access difficulties (declared by the patient) and perceived difficulties anticipated by the health professional (van Loon et al., 2019). To assess the real impact and relevance of the A-DIVA scale, its systematic and isolated implementation will be carried out in phase 2 of the ALCOV study. The results obtained will guide the determination of which criteria could benefit caregivers in solving the difficult vascular access problem.

Two hundred and forty-five patients were successfully punctured on the first attempt, representing an 82% first-attempt success rate; 12% had successful puncture on the second attempt; 6% had successful puncture on the third or subsequent attempt. Our results thus highlight the major health concerns raised by difficult vascular access, since failures pose a risk to patient safety and to the well-being of health professionals at work, leading to a loss of self-efficacy and feelings of incompetence and making it difficult to address patient distress (qualitative results of the study).

In cases of difficulties, health professionals have implemented strategies to cope with failure, mainly by calling another professional, using an alternative puncture technique, or choosing another type of vascular access. However, in one-third of the cases, no strategy was implemented. During the interviews, health professionals emphasized the lack of specific protocols and called for them. These data fully justify the need for the development of an algorithm, the establishment of a formal organization, and the generation of specific protocols to guide health professionals when dealing with difficult vascular access in contexts where they have restricted access to alternatives to traditional insertion by visualization/palpation (e.g., ultrasound-guided insertion or transillumination).

The qualitative analysis revealed that there was no consensus among health professionals regarding the incidence of difficult vascular access, with many patient profiles being described as potentially at risk. This highlights the subjective and perceptual nature of the phenomenon (Armenteros-Yeguas et al., 2017; Bahl et al., 2021; Civetta et al., 2019; Ng et al., 2022).

The variations in peripheral venous catheter performance linked to individual factors (stress, fatigue, emotional state, etc.) were outlined. These factors are recognized by health professionals as inherent to the insertion process (i.e. non-linear individual performance of the gesture). Multiple tips to anticipate failure have been developed by health professionals, with each one developing these tips at their own level.

The results also revealed that there was an unspoken rule among health professionals that stated that, after two failures, the peripheral venous catheter should be handed over to another health professional. This idea is consistent with findings from other studies and can be explained by the desire to preserve the patient’s venous capital; it also supports the notion that aggressive treatment is counterproductive (Bahl et al., 2021; Ng et al., 2022; Stagg, 2023) (see the “Tips and areas where improvements can be made” results section). However, mutual support and non-judgement between health professionals emerged as the norm in the departments.

Health professionals clearly emphasized the need for best practice guidelines to be implemented for peripheral venous catheter placement in departments to optimize overall patient care. The development of a decision-making tree tailored to puncture conditions (algorithm) to manage patients undergoing peripheral venous catheter placement should include these individuals and collaborative levels (intra- and inter-health professionals).

Our data also suggest that an organizational reflection should be conducted, since failures could also be caused by factors related to the health care setting:

(I) Working conditions such as night shifts, weekends and understaffing do not always allow teamwork that enables handover to another health professional if necessary.
(II) Workspaces can hinder peripheral venous catheters (which is important to consider when identifying puncture conditions).
(III) Difficulties in anticipating workload can cause stress, which can affect health professionals’ performance.
(IV) Nurses expressed a desire for greater access to training in alternative techniques, such as transillumination and ultrasound. However, they lack knowledge of their rights to practice. Note: In France, nurses can access these techniques after completing a certified training course, provided that their hospitals fulfill the requirements set out in a document forwarded to the Regional Health Authority (“Arrêté du 1er mars, 2021 relatif à l’autorisation du protocole de coopération « Réalisation d’échographies des veines et/ou artères des membres supérieurs par une infirmière en lieu et place d’un médecin » - Légifrance,”)

Our set of quantitative and qualitative data will be submitted to four working groups in the next phase (II) of the project. The data will inform the construction of an algorithm for risk assessment and the development of safe, effective and stratified corrective actions, considering the specifics of each punction situation within conditions of a health care setting.

In our study, we found that 18% of the peripheral venous catheters required at least two punctures. Given the objective of reducing the average number of punctures per patient, it seems reasonable and clinically relevant to aim to reach a 15% decrease in phase III with the development and implementation of the algorithm for peripheral venous catheter placement. Moreover, a step-by-step implementation process is used in the global ALCOV project to ensure the transferability of the intervention on a large scale for emergency departments and operating theaters (Skivington et al., 2024).

### 4.1 Limitations

The main limitation of this study is that the health professionals at the two selected centers were experienced in peripheral venous catheterizations due to frequent vascular access placements. This is probably why their performance was overrated in comparison with centers where professionals were less exposed to peripheral venous catheters. This choice ensures sufficient recruitment to assess the effectiveness of the intervention. However, the potential for bias will require careful consideration of how to sustain large-scale deployment of ALCOV to operating theatres and emergency departments where health professionals are slightly less experienced. Moreover, during Phase I, low pain thresholds were observed. This finding could also be linked to the great expertise of the health professionals involved. As pain assessment is not a standard practice when placing peripheral venous catheters, this measurement was not mandatory in the present study to avoid changing current practices.

## 5 Conclusions

Our findings reveal critical gaps in current peripheral venous catheter placement, particularly the absence of standardized placement protocols and the influence of contextual and emotional factors on procedural outcomes. The organization of peripheral venous catheter placement care will be defined by considering the following factors: (i) individual factors related to patients and health professionals, (ii) collaborative factors, and (iii) contextual factors related to the particular health care setting. Key factors identified in Phase I of the ALCOV project will enable the development of a comprehensive, consensus-based algorithm for the placement of vascular accesses. This will be followed by the risk-score impact analysis in Phase II and the groups' work during the interphase. Then, Phase III will involve implementing this algorithm and evaluating its impact. Each step, essential to a thoughtful development process, will be described in the near future.

## Data statement

The quantitative data analyzed in the current study are available from the corresponding author upon reasonable request. Full participant transcripts have not been included in the manuscript because the data contain potentially identifying or sensitive information. If researchers wish to work on our data, we will proceed with a confidentiality agreement drawn up under the supervision of our local data protection lawyer. Contact information to which data requests may be sent to the corresponding author.

## Funding source information

This study was supported by a grant from the French Eastern Interregional Group of Clinical Research and Innovation (GIRCI Est) (Appel à Projet PARAmédical, APPARA 2022) and by CHRU de Nancy.

## CRediT authorship contribution statement

**Laetitia Ricci:** Writing – original draft, Validation, Supervision, Methodology, Investigation, Funding acquisition, Formal analysis, Conceptualization. **Florian Manneville:** Writing – original draft, Validation, Supervision, Methodology, Funding acquisition, Formal analysis, Conceptualization. **Côme Slosse:** Writing – original draft, Validation, Supervision, Methodology, Investigation, Funding acquisition, Formal analysis, Conceptualization.

## Declaration of competing interest

The authors declare that they have no known competing financial interests or personal relationships that could have appeared to influence the work reported in this paper.

## Appendix Supplementary materials

Image, application 1
