# Supplementary material for: Reality and challenges of peripheral vascular access: a mixed-methods study
Source: Int J Nurs Stud Adv. 2026 May 7;10:100554. doi: 10.1016/j.ijnsa.2026.100554 (PMC13185776; doi:10.1016/j.ijnsa.2026.100554)
Supplement: Supplementary file 1 [file mmc1.docx]

**Semi-structured individual interview guide for professionals**

Theme 1:

- Can you describe the characteristics of the difficult vascular access (DIVA) situations you encounter in your daily practice?

1. ....

2. ....

3. ....

4. ....

5..... Etc.

- Can you think of others that are less frequent?

Note: the interviewer will fill in the situations and investigate the practices of the careers for each situation encountered, which corresponds to the meaning of the explanatory interview (breaking down the structure of a complex task in a precise rather than a general way).

Theme 2:

- For each situation, can you describe precisely what you are doing and with whom (alone, in pairs, in trios, etc., and how the tasks are divided up)?

- What tips and tricks do you use (risk anticipation strategies) to manage these situations?

- For each situation, what difficulties might you encounter?

Theme 3:

- How do you feel you are supervised in the management of DIVA in your department?

- How do the care procedures in force in the department impact DIVA practices?

Theme 4:

- How does the organization of the department affect the way DIVA is managed? (For example: staff present (number and duties/qualifications), position and working hours, presence of a student, etc.)?

Theme 5:

- Think about the place where you work most often, if it had to be reorganized to make it easier to deal with DIVAs? What would you do?

- What do you think of the type of equipment available to you? To what extent do you consider this equipment quantitatively sufficient?

Theme 6:

- Have you ever taken any training courses in this area? If so, in what context? Moreover, what did they teach you? Have you done any other research? If so, how, why and what did you learn?

Theme 7:

- Are there any things you would like to do now but cannot?

Theme 8:

- Are there any other things you would like to tell me about your DIVA practices?
